# The Influence of Cone Age and Urbanisation on the Diversity and Community Composition of Culturable Seed Fungal Endophytes within Native Australian *Banksia ericifolia* L.f. subsp. *ericifolia*

**DOI:** 10.3390/jof9070706

**Published:** 2023-06-27

**Authors:** Merize Philpott, Edward C. Y. Liew, Marlien M. van der Merwe, Allison Mertin, Kristine French

**Affiliations:** 1 Centre for Sustainable Ecosystems Solutions, School of Earth, Atmospheric and Life Sciences, University of Wollongong, Wollongong, NSW 2522, Australia; kris@uow.edu.au; 2 Research Centre for Ecosystem Resilience, The Royal Botanic Gardens and Domain Trust, Mrs Macquaries Rd, Sydney, NSW 2000, Australia; edward.liew@botanicgardens.nsw.gov.au (E.C.Y.L.); marlien.vandermerwe@botanicgardens.nsw.gov.au (M.M.v.d.M.); allison.mertin@botanicgardens.nsw.gov.au (A.M.)

**Keywords:** community composition, diversity, endophyte, mycobiome, seed, seed fungal endophytes, temporal changes, urbanisation, *Banksia ericifolia*

## Abstract

Seed fungal endophytes play a crucial role in assisting the overall health and success of their host plant; however, little is known about the factors that influence the diversity and composition of these endophytes, particularly with respect to how they change over time and within urban environments. Using culturing techniques, morphological analyses, and Sanger sequencing, we identified the culturable seed fungal endophytes of *Banksia ericifolia* at two urban and two natural sites in Sydney, New South Wales, Australia. A total of 27 Operational Taxonomic Units were obtained from 1200 seeds. Older cones were found to contain, on average, more colonised endophytes than younger cones. Species richness was also significantly influenced by cone age, with older cones being more speciose. Between urban and natural sites, the overall community composition did not change, although species richness and diversity were greatest at urban sites. Understanding how these endophytes vary in time and space may help provide an insight into the transmission pathways used and the potential role they play within the development and survival of the seed. This knowledge may also be crucial for restoration purposes, especially regarding the need to consider endophyte viability in ex situ seed collection and storage in seed-banking practices.

## 1. Introduction

Discovering the hidden and diverse world of plant-associated microorganisms has provided fundamental insights into their ecological significance for plants. Plants harbour both epiphytic and endophytic microbial communities in all their tissue types [1,2]. The actions of these microorganisms, particularly endophytes, may significantly influence plant traits and responses, including tolerance to drought and disease [3,4,5,6], the improvement of carbon fixation [7], and metabolite production [8], suggesting that endophytes may ameliorate the effects associated with abiotic and biotic stressors. To date, most research has focused on the composition and structure of the microbiota associated with the roots, leaves, and stems, while very few studies have concerned fruit and seeds [8]. Seeds generally have high mortality rates [9,10] due to granivores, fungal pathogens, and exposure to environmental stressors [10,11,12,13,14]. Seed endophytes can exist as mutualists, as latent pathogens that may express disease symptoms over time, or as saprotrophs that are active during tissue senescence [15,16,17,18]. Endophyte colonisation within seeds can enhance seed germination [19,20] but may also influence seed survival through deterring herbivores [21] and even stimulating early seedling growth [22]. The lack of research into factors that affect seed endophyte composition and diversity hinders our understanding of the breadth of the impact that these factors may have on individuals and the regeneration of whole plant communities. This is particularly important for plants containing seed-borne endophytic species such as *Epichlöe* spp., which are primarily found in grasses [21]. Investigating variation in seed endophytes may allow for the more successful management and monitoring of conservation practices [23].

Plants acquire fungal endophytes through two main pathways: vertical transmission from the maternal host plant via the seed, and horizontal transmission from the surrounding environment such as the soil, dead leaf material, or aerosols [24]. Although the relative importance of these transmission methods remains unclear, both modes contribute to the overall composition of the plant microbiome, including the seed microbiome [8]. Colonisation is likely mediated by the environment in which the host exists, although the corresponding lack of information and equivocal results in this regard hinder the development of a general framework for colonisation. Some work suggests that fungal endophytes colonise hosts in greater abundance within nutrient-poor environments [25], with a decrease in endophyte richness in leaves containing higher nitrogen-to-phosphorous ratios [26]. However, Hamilton et al. [27] showed that the abundance of a non-hybrid endophyte species increased with an increase in the number of soil nutrients, whereas a hybrid (hyphal anastomosis between two fungal species) endophytic species in the same host was positively associated with increasing soil moisture and pH levels.

The age of plant tissue potentially plays a significant role in the diversity and composition of fungal endophyte communities [28]. Currently, the results from different studies are not congruent, with some showing an increase in diversity over time [29,30,31,32] with a decrease in abundance [32], while other indicate the opposite, presenting higher richness and diversity in younger tissues [33]. This discrepancy may be a result of different foliar physiologies associated with the availability of different nutrients or a result of differences in the specificity of species to endophytic species [33]. It may also reflect the mode of arrival of endophytes into the seed in different habitats. Although most studies have concerned endophyte movement within leaves or roots, we have no information about the temporal changes in the seed microbiome outside of ex situ storage.

Urbanisation has increased significantly over the last 30 years [34], impacting local abiotic conditions, including via increases and decreases in soil nutrients, air pollution [35], higher heat loads, chemical contamination, and the compaction of soils [36]. Seed fungal endophyte colonisation may be significantly influenced by these factors, which may filter out certain species that are not preadapted or cannot tolerate the physiochemical conditions created [37]. However, these changes may also allow some endophytic species to colonise due to the increased stress in the host plant, a reduction in host defences, or the presence of wounds. To date, little is known about changes in microbial communities within urban environments [38], although there has been a recent influx of studies [39,40,41]. Urban soil studies have shown differing responses of fungi and bacteria to urbanisation [42], with fungal diversity generally decreasing [42,43,44,45] and bacterial diversity increasing or remaining unchanged [40,42,45,46]. Local site conditions may play a significant role in the diversity and composition of fungal communities [47], and the characteristics of urban sites may, therefore, change fungal community composition. In remnant vegetation in the Japanese city of Chiba, there was a large decline in host-specific fungal species and an overall decrease in the diversity of the community [48]. A similar pattern was also found in urban areas in Finland [43], while Klaedtke et al. [47] found a strong local site effect on the assembly of seed fungal endophytes in Brittany and Luxembourg, Europe.

The consideration of the seed microbiome and its role in longevity, germination, and early growth is crucial for a variety of conservation actions. This is particularly important considering that endophyte viability decreases in seed banks [49], which potentially reduces the success of therestoration practice. For species stored in seed vaults, it is important to understand their age-related ecology to ensure that all aspects of the plant are captured for its future survival and re-introduction. It is also important to consider the conservation success of using seeds stored in canopies, which lose viability with age and are faced with increased levels of herbivory and seed decay [50].

Studying a serotinous seed bank provides opportunities to gain insights into the colonisation pathways of seed fungal endophytes [51]. The storage of seeds within maternally derived woody follicles is likely to bring about both environmental transmission, including with regard to various saprotrophic species that colonise woody substrates [52], and maternal transmissional pathways during foliar development. High variation in the assemblage of seed endophytes amongst seeds in young follicles from different sites would suggest that environmental pathways are important in the early stages of development. Low variation would suggest one of three options: a filtering mechanism of environmental transmission via maternal tissue, a fundamental species-specific assemblage transferred from the mother, or a ubiquitous suite of spores transferred environmentally.

Each of these possibilities predicts a different outcome with respect to what happens as these seeds age. The observation of similarities between young and old seeds at a site would suggest that environmental and maternal transmission is unlikely in these woody cones once fully developed. There is no doubt that the woody follicles surrounding seeds act as a barrier to many microbes, but they may not stop all species from colonising. Differences in endophyte assemblages between young and old cones would suggest one of three options: environmental transmission is occurring through the woody infructescence; there is competition amongst endophytes within seeds, leading to assemblage homogenisation; or there is a further transfer of endophytic species from maternal tissue. If competition is occurring, then older seeds should become more similar in composition across sites, even if they started out being quite different. A ubiquitous set of endophytes that are environmentally transmitted should result in little change with age and few site-to-site differences. Furthermore, the comparison of seeds from sites that differ in disturbances, such as urbanisation, is likely to further implicate the role of environmental transmission, as site characteristics ought to influence the species that are transmitted. Therefore, in this study, we aimed to determine the influence of seed age and urbanisation on the diversity and composition of culturable seed fungal endophytes within a serotinous species, namely, *Banksia ericifolia* L.f. subsp. *ericifolia*.

## 2. Materials and Methods

### 2.1. Study Species and Sites

In Australia, *Banksia* species (Proteaceae) form an abundant part of the natural scleromorphic vegetation found on the eastern coast of the continent [53]. A multi-flowered inflorescence forms a fertile, woody infructescence upon pollination [54], storing seeds within closed follicles in the tree until their release is triggered by fire [55]. Serotinous seeds gradually lose viability with age, with seed decay more evident in older seeds [50]. *Banksia ericifolia* ssp. *ericifolia* is predominantly found in coastal heathland on shallow sandstone in the Sydney bioregion [56]. It relies on the release of seeds following plant death from fire for the production of the next generation of individuals [57]. The plants cannot resprout post-fire and rely on seeds for regeneration.

### 2.2. Seed Collection

**Figure 1:**
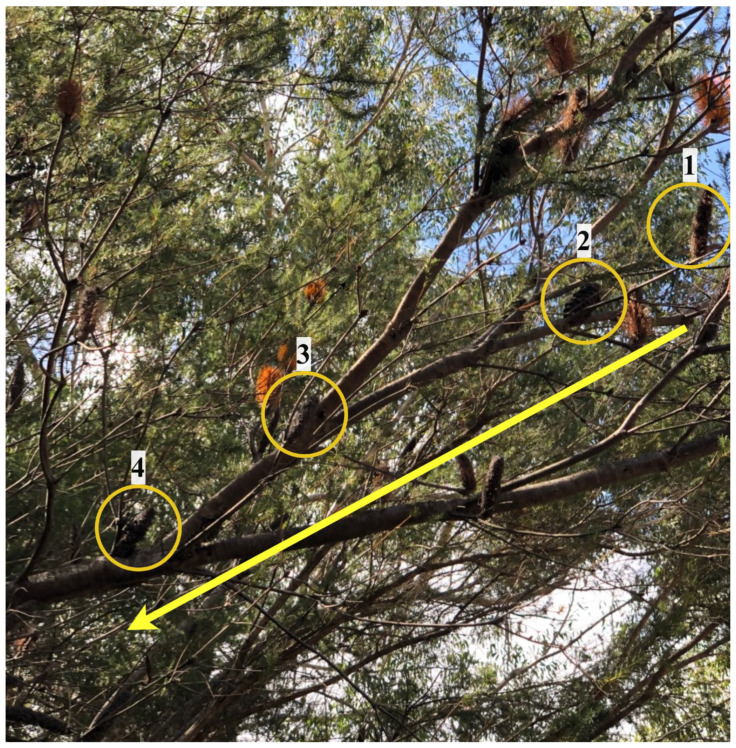
The cone age of *Banksia ericifolia* was estimated based on the annual infructescence formation at each node between two branches. The newest cases of infructescence, highlighted by the yellow circle with the number one, are located on the nodes furthest away from the base of the tree. The yellow arrow points toward the base of the tree; cone node formation becomes more distant in time when moving toward the base, where the oldest cones on the tree are located. In this image, the oldest cone is located within the yellow circle highlighted by the number four.

Two heathland sites, one located within the Royal National Park (RNP) (−34.13° S, 151.08° E) and the other within Patonga in Brisbane Waters National Park (PTG) (−33.53° S, 151.28° E), were selected for seed collection as both contain natural vegetation that is relatively undisturbed by anthropogenic influences. Seed collection occurred in September and October 2020, respectively. Average seasonal rainfall values in RNP and PTG were 8.5% and 5.0% lower, respectively, than normal, with an average of 65 mL in RNP and 75 mL in PTG. The average temperature in Spring is 21 °C in RNP and 22 °C in PTG. The estimated age of each tree in both sites was over 25 years old. The last fire in RNP was in 1994, while the last fire in Patonga was in 1990–1991. The age of each seed was estimated based on the annual growth pattern of each infructescence, with the youngest and newest cones for the year located at the newest node of growth. Each node closer to the main trunk represents an earlier year of growth and reproduction. Therefore, the age of the seeds could be estimated (Figure 1). Old cones were defined as those that were greater than eight years old, while young cones were defined as those less than four years old.

Two remnant patches of dry sclerophyll forest on shallow sandstone soils were identified as urban sites, namely, Lane Cove National Park (LNP) (−33.75° S, 151.11° E) and Kamay Botany Bay National Park (BNP) (−33.98° S, 151.24° E), as they were within 15 km of the Sydney Central Business District. Both sites contained an abundance of young (less than four years old) *Banksia ericifolia* and evidence of anthropogenic influence. Urban seed collection occurred in February 2021. The average summer rainfall amounts during the collection period at LNP and BNP were 21.4% and 30.5%, respectively, lower than normal, with approximately 88 mL recorded in LNP and 98 mL recorded in BNP. The average summer temperatures are 27 °C in LNP and 26 °C in BNP.

At each site situated in natural areas (500 × 500 m), old and young cones were collected from different trees. Four transects were set up (100 m apart), from which ten trees (five per transect) were selected to sample young cones approximately every 100 m along two of the transects using the line transect intercept method [58]. Ten trees (five per transect) were also chosen for the collection of older cones on the other two transects. Within urban areas (500 × 500 m), two transects were constructed 100 m apart, from which ten trees (five per transect) were selected approximately every 100 m along using the line transect intercept method [58], with only young cones collected from different trees. From each of the selected trees, three mature woody infructescences containing seeds were randomly collected and visually compared with respect to signs of disease and insect damage. Upon final collection, infructescences were stored at room temperature with their seeds extracted (see below) and plated onto agar plates within three days of collection.

### 2.3. Seed Endophyte Extraction

Woody infructescences were cleaned by hand to remove anthers and washed using tap water to remove dirt and any insects. Following cleaning, each cone then had a propane pinpoint gas torch (Tradeflame, Australia) [51] directed onto ten randomly selected follicles for 20 s until a glossy surface was observed and follicles were opened,; exposing the seeds. The exposed seeds were then extracted using tweezers, and viable seeds (i.e., seeds containing fleshy endosperm and no signs of insect damage or decay) were kept for endophyte isolation. Ten viable seeds were immediately surface-sterilised in a laminar flow using 70% ethanol by spraying both sides of the seed until covered; then, they were left for 30 min to air dry. An initial test was conducted to test the efficacy of this sterilisation technique by imprinting the sterilised seeds onto an agar plate, for which the same growth conditions as used in this study (see below) were followed. No endophytic growth was identified.

Seed fungal endophytes were isolated by plating each of the surface-sterilised seeds (1200 seeds in total) onto a separate 60 mm petri dish containing 1/4 strength potato dextrose agar (PDA) with added 2% lactic acid to suppress bacterial growth. Each plate was placed into an incubator in the dark at 22 °C for 28 days and examined for endophyte growth every 7, 14, and 28 days to ensure the capture of both fast- and slow-growing species. To obtain pure endophyte colonies for DNA extraction, the colonies grown at each of the three time points were sub-cultured onto Carnation Leaf Agar (CLA) via hyphal tipping using the protocol reported by Davey et al. [59] and incubated on a light rack (Philips Cool Daylight, 36 W/865), which mimics natural levels of ultraviolet radiation to encourage fungal sporulation.

### 2.4. DNA Extraction and Sequencing

**Figure 2:**
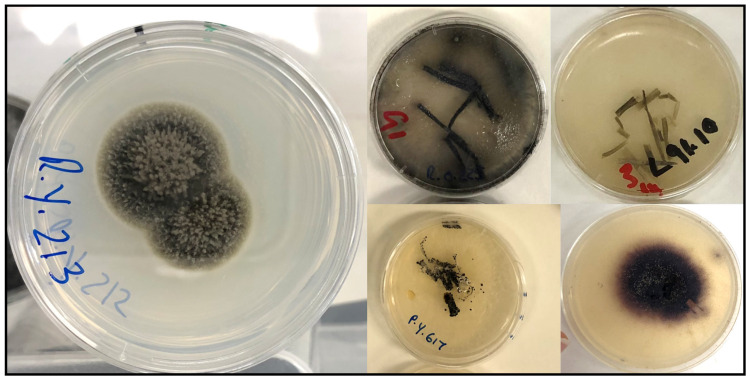
Separating seed fungal endophyte cultures on CLA using morphological analyses.

Following the incubation of endophytes on CLA, fungal cultures were grouped based on similarities in colony morphology, including form, colour, surface (including fuzziness), shape, and elevation, following the methods described by Silva et al. [60]. A subset of samples from each morphotype was selected for DNA extraction and analysis (Figure 2). To identify the seed fungal endophytes present, genomic DNA was extracted from the mycelium using the DNA FastPrepTM Kit (Q-biogene Inc., Carlsbad, CA, USA) as per the manufacturer’s instructions.

Following successful DNA extraction, ~500 base pairs of the internal transcribed spacer region (ITS) of each fungal culture were amplified using the primers ITS1F/ITS4 via polymerase chain reaction (PCR) and Sanger-sequenced in the forward direction following the same procedure and conditions as those reported by Mertin et al. [51]. Capillary electrophoresis was also performed at Ramaciotti Centre for Gene Function Analysis, Australia, using an ABI PRISM^®^ 3730 DNA Analyzer (Applied Biosystems Inc., Foster City, CA, USA). The quality of the DNA samples was assessed using the high-quality score (HQ%) index in Geneious v11.0.9 with a 70 HQ% threshold cut off. The sequences were manually trimmed, and electropherograms were checked for missed nucleotide base calls.

Operational Taxonomic Units (OTUs) representing 97% sequence similarity were identified using the programme CD-Hit [61], with the input sequence dataset comprising 500 bp of partial 18S, ITS1, 5.8S, ITS2, and partial 28S regions. Species identities were then assigned to each of the OTUs, with the ITS sequences downloaded from NCBI’s GenBank database, and species names were assigned to each OTU from which a species was most closely clustered to in the genus phylogeny. The samples clustered into OTUs were then compared to the fungal cultures in the original morphotype analysis to confirm successful identification. Representative cultures were submitted to GenBank with the resultant accession numbers listed in Supplementary materials (Table S1).

### 2.5. Data Analyses

To determine whether the proportion of follicles colonised per tree and the abundance of OTUs per tree differed with seed age (less than 4 years or greater than 8 years) and site (Royal National Park and Patonga), a two-factor ANOVA was performed (JMP v15; SAS Institution Inc, Cary, NC, USA, 1989). Tukey’s HSD tests were used where necessary to determine differences.

To determine whether the seed fungal endophyte assemblages differed with age and site, a two-way permutational multivariate analysis of variance (PERMANOVA) test with 999 permutations was conducted in Primer-e v7 [62] using Bray–Curtis similarities. Following bootstrapping of the averages for each site according to age group (75 pseudoreplicates), the differences in the endophytic fungal communities between site and age were visualised using metric multidimensional scaling (mMDS) with a 95% confidence interval in Primer-e v7 [62].

To investigate differences in communities occupying trees in urban and natural areas, a nested two-way PERMANOVA was conducted in Primer-e V7 [62], with sites nested in habitats. To determine whether the proportion of seed follicles colonised and the number of OTUs per tree differed with habitat (urban versus natural), a nested ANOVA analysing sites within a habitat was conducted, followed by Tukey’s HSD test to determine differences. A square root transformation improved normality. Potential fungal guilds were assigned using the FUNGuild database query tool [63] based on species names. In addition, literature records were searched to support the FUNGuild assignment [64,65,66,67,68,69,70,71,72,73,74,75,76,77,78,79,80,81,82,83,84,85].

## 3. Results

### 3.1. Effect of Cone Age on Endophyte Assemblages

**Figure 3:**
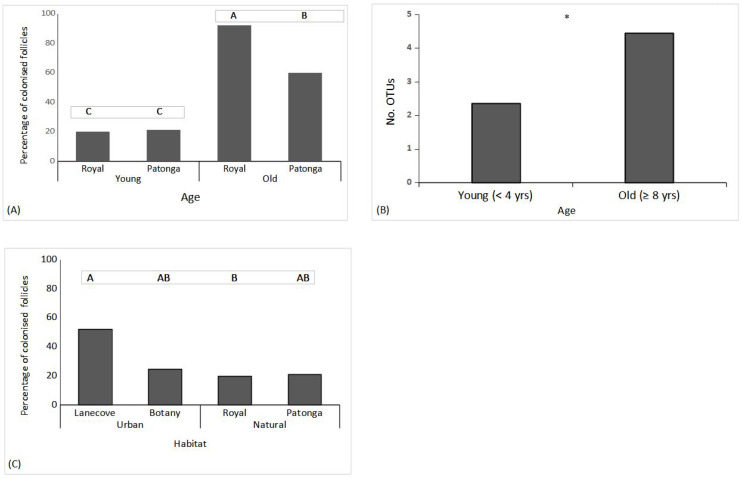
(**A**) Average percentage (+se) of colonised follicles of cones of different ages for Banksia ericifolia trees at Royal National Park (Royal) and Patonga, and (**B**) average number of OTUs per cone within 20 young (less than four years old) and 20 old (greater than eight years old) trees in natural areas (Patonga and Royal National Park). (**C**) Average percentage (+se) of colonised follicles for Banksia ericifolia trees in urban sites (Lane Cove National Park and Kamay Botany Bay National Park) and natural sites (Royal National Park and Patonga). Similar lettering (capitalized) indicates differences between means in separate Tukey’s HSD tests for each graph. The asterisk indicates where differences were found via ANOVA.

A total of 674 endophytic isolates belonging to 27 OTUs were obtained from 1200 seeds. From RNP, 399 isolates were obtained, while 275 isolates were from PTG. Across both sample sites, 38 out of 40 plants contained at least one fungal endophyte; 2 trees within RNP had no isolates from young seeds. Analysis of the proportion of endophytes colonised within seed follicles indicated that the effect of age on endophyte colonisation was influenced by site (F(1,36) = 9.652, *p* = 0.004, Figure 3A). For both sites, older cones contained more endophytes, with the plants from RNP having a three-fold greater proportion of follicles with endophytes compared to young follicles and the PTG plants having at least double this measure. Young cones from both sites had equivalent colonisation levels (Figure 3A). Across both sites, a total of 27 OTUs were detected based on the clustering of ITS sequences at 97% sequence similarity. Regardless of the site, older cones contained nearly double the OTU richness of the younger cones (F(1,36) = 23.70, *p* < 0.001) (Figure 3B).

**Figure 4:**
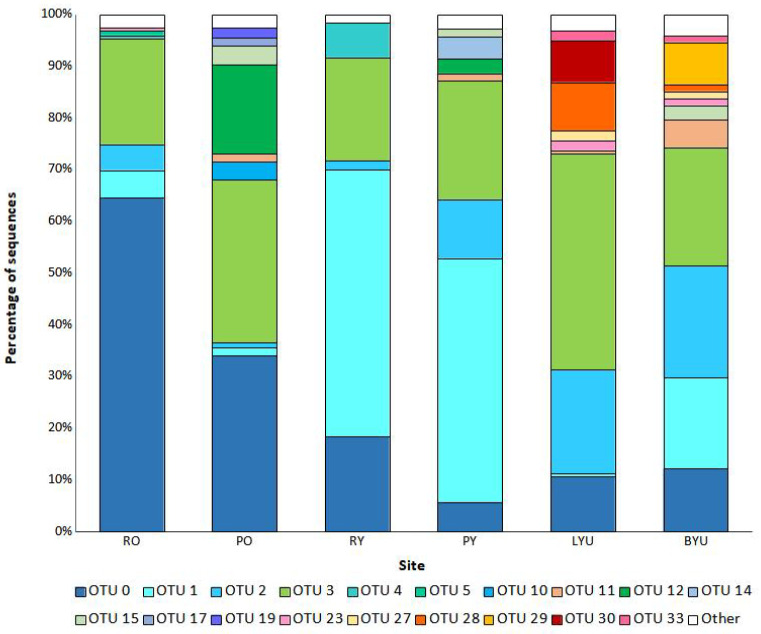
Relative abundance of operational taxonomic unit (OTU) sequences calculated as a percentage of total Internal Transcribed Spacer (ITS) sequences assigned for each site. R = Royal National Park, P = Patonga, B = Kamay Botany Bay National Park, L = Lane Cove National Park, Y = Young (less than four years old), O = Old (greater than eight years old), and U = Urban.

The most abundant OTU, OTU_0 (containing 290 isolates), was identified as *Penicillium citreonigrum* and was predominantly found within older individuals (275 isolates) (Figure 4, Table 1). The second most abundant OTU, OTU_3 (containing 156 isolates), was identified as *Banksiamyces* sp. and was also most prominent in older individuals (128 isolates). Collectively, the sequences belonging to both OTU_0 and OTU_3 constituted 64% of all the sequences. In contrast, OTU_1 (containing 64 isolates), which was identified as *Neofusicoccum* sp., was most abundant in younger individuals. Across both sites, both young and old trees housed seed fungal endophyte (SFE) communities that consisted of a few highly abundant species and many rare species, with a total of 12 OTUs detected within young cones and 24 OTUs in older cones, for which there were 9 species in common (Figure 4).

**Figure 5:**
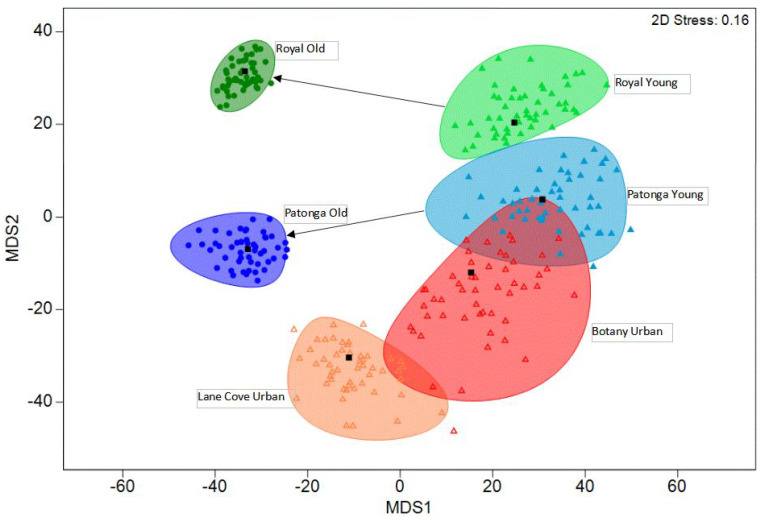
Multidimensional scaling ordination (metric) of group means of the composition of endophytes in seeds from trees of *Banksia ericifolia* using bootstrapping (bootstraps per group = 75) and smoothed regions covering 95% of bootstrapped averages. Bray–Curtis similarities of abundance data were used. At two natural sites, namely, Patonga and Royal National Park, old cones (greater than eight years old) and young cones (less than four years old) were sampled from independent trees. At two urban sites, namely, Lane Cove and Kamay Botany Bay, only young cones were collected.

While SFE compositions differed with age, the differences were site-specific (interaction term, PERMANOVA: Table 2). The assemblage of endophytes in the seeds from older cones on trees were distinct from the assemblages in the trees where younger cones were collected (RNP t = 3.94, *p* = 0.001; PTG t = 2.93, *p* = 0.001). Trees with younger cones from the two sites had a similar assemblage (t = 0.96, *p* = 0.50), while the sites were different in the assemblage of endophytes from the trees with older cones (t = 3.71, *p* = 0.001) (Figure 5). Furthermore, there is a noticeable degree of similarity amongst assemblages in the old cones (small ellipses), with higher variability evident in the assemblages of younger cones.

### 3.2. Comparison between Urban and Natural Areas

Within the urban habitats, a total of 234 endophytic isolates belonging to 20 OTUs were obtained from 600 seeds. A total of 160 isolates were obtained from the seeds of LNP, and 74 isolates were obtained from BNP. In contrast, 130 endophytic isolates belonging to 12 OTUs were obtained from 600 young seeds collected in the natural habitats. A total of 60 isolates were obtained from the seeds from RNP, and 70 isolates were obtained from PTG in Brisbane Waters National Park. Across both urban habitats, 18 out of 20 trees contained at least one fungal endophyte.

The effect of habitat type on the proportion of endophytes isolated was influenced by the site from which the samples were collected (F(2,36) = 3.410, *p* = 0.044) (Figure 3C). Overall, for urban habitats, LNP contained the highest average colonisation level, with trees having more than double the proportion of isolates compared to other sites (Figure 3C).

Within urban sites, the most abundant OTU, OTU_3 (84 isolates), was identified as *Banksiamyces* sp. (Figure 4). The second most abundant OTU, OTU_2 (48 isolates), was identified as *Penicillium glabrum*. Collectively, sequences belonging to both OTU_3 and OTU_2 constituted 73.8% of all the sequences within urban sites. In contrast, the most abundant OTU found within natural sites, OTU_1 (64 isolates), was identified as *Neofusicoccum hellenicum*, which constitutes 70.61% of all sequences within natural sites. Urban sites contained a range of unique species with 11 OTUs unique to urban sites (Table 1). The ordination of the endophyte assemblages showed similarities amongst the young cones (Figure 5). The overall SFE community composition between the urban and natural sites did not change significantly (Table 2).

## 4. Discussion

Over time, the retention of *Banksia* seeds within the tree canopy over multiple years [86] allows for the horizontal transmission of endophytes into seeds due to the large dispersal ability of fungal spores [43]. As cones aged in the natural areas of our study, there was a significant and site-specific change in the assemblage, which is indicative of the arrival of new endophytes through horizontal transmission despite the woody infructescence, which potentially acts as a filter.

Our study provided evidence of increasing environmental transmission of seed endophytes over time. Firstly, the colonisation and richness of fungal endophytes detected within older seeds were nearly double those from the younger seeds, suggesting that a range of fungal endophytes arrived and colonised as the seed matured, with the infructescence forming woody tissue and the seeds being held in the canopy for multiple years (even decades). Secondly, the assemblage was quite different and diverged across the sites, suggesting that site-specific factors were influencing colonisation. While the idea that endophytes accumulate with tissue age has been proposed in many studies [87,88,89], these concepts have been developed for leaf age or root development over time. Species richness was doubled in old leaves in comparison to young leaves in *Banksia integrifolia* [90], and similar increases were found in seven mangrove species in south India [91], although other studies have shown a decrease in endophyte richness in coastal redwood trees in California [92] or no difference with foliar age [93]. In our study, it was determined that fungal spores may be able to penetrate through the woody barrier of *B. ericifolia* cones in order to enter the seeds, perhaps seeping in during and after rainfall events. The identification of endophytes in maternal tissues supporting infructescenses would provide insights into whether there is any transmission via maternal support through xylem and woody tissue or whether transmission is associated with fungal spores breaching the sealed follicles.

Our study also showed patterns suggesting that there are both selective and competitive behaviours relating to the overall composition of seed fungal endophytes within young seeds. The sites where young seeds were sampled (both urban and natural) had similar assemblages and were dominated by *Penicillium citreonigrum, Banksiamyces* sp., *Neofusicoccum hellenicum*, and *Penicillium glabrum*, suggesting that there is potentially some selective transfer of fungi from the maternal plant into the seed during development. This core suite of four species was present at all sites and persisted through time, although they changed in terms of frequency of occurrence. The presence of these species may be selected for their saprotrophic lifestyle, in which they assist in providing the germinating seed with nutrients upon release after fire. This may be particularly important in the earlier stages of seed storage, wherein seed viability is highest. Within these early stages, horizontal transmission may still be occurring, with species competition shaping the composition. The presence of *Banksiamyces* sp., particularly within the seeds, supports the initial idea of the selective maternal transfer of some endophytic species. Species of this genus can exist endophytically within the seed and switch to a saprotrophic lifestyle within dead *Banksia*, producing fruiting bodies on the cone follicles [94]. This lifestyle classifies this species as a Class 2 nonclavicipitaceous endophyte [95], whose members are known to be vertically transmitted via the seed coat [96]. As the seeds aged and their viability began to decrease, a shift in the community composition toward endophytic species with the ability to support seed survival was observed.

Over time, the changes in the abundance of these commonly occurring species may occur through new colonisations via either maternal or environmental transmission or via competitive interactions causing changes to the resident assemblage. *Penicillium citreonigrum* was more highly abundant in older individuals compared to *Neofusicoccum hellenicum*, which was more abundant in younger individuals. The pronounced presence of *Penicillium citreonigrum* within older *B. ericifolia* may influence herbivory, as this species produces citroviridin, which deters mammalian herbivores [97]. This is particularly important, as *Banksia* seeds are retained within the canopy for many years and are, therefore, susceptible to high levels of predation and decay [50]. The presence of *Penicillium* within both young and old seeds may be advantageous, as it is known to increase seed germination in *Phragmites australis* [98]. The genus *Neofusicoccum* has been found in a wide range of hosts as an endophyte but may change to a pathogenic phase during periods of stress and may result in disease symptoms such as cankers and dieback, which can lead to plant death [99]. *Neofusicoccum* is relatively fast growing, a strong competitor for resources [100], and it predominantly spreads via spores to new hosts [99]. The abundance of this species within young cones highlights the vulnerability of young seeds to the horizontal transmission of particularly pathogenic species during seed development and storage. Therefore, decreasing abundance of *Neofusicoccum* within older cones may be associated with increased competition with late-arriving or slower-growing species. Although many endophytes such as *Penicillium* and *Fusarium* species have been shown to be beneficial to the host, they have also been identified as latent pathogens [17,51]. The presence of latent pathogens within this study highlights the need for more SFE studies in order to understand disease spread and the future conservation of the species.

Continued horizontal transmission may explain the presence of a few introduced pathogenic and saprotrophic species within the seeds from the urban sites. These species may have been introduced through the movement of spores initially transported to the sites by domesticated pets and feral birds or from the gardens of exotic flora species near the sites [101]. The presence of the pathogen *Phaeocremonium scolyti* at both LNP and BNP accentuates the need for further monitoring programs, as this species is associated with stunted plant growth and dieback in woody plants and with opportunistic human infections including phaeohyphomycosis [102]. Also isolated from both urban sites, the saprotrophic fungus *Exophiala oligosperma* is commonly found in domesticated hot and humid environments with low nutrient availability [103] and in areas that are rich in hydrocarbons [104,105]. This species is also commonly associated with human skin and subcutaneous infections including onychomycosis [105]. The presence of both species within urban sites highlights the negative impacts of anthropogenic influence on urbanisation and the introduction of pathogenic species, placing strain on both the health of the ecosystem and of humans.

The colonisation processes that occurred in the analysed natural sites (Royal National Park and Patonga) appeared largely intact in urban national parks. Our study showed that seed fungal endophyte communities within *Banksia ericifolia* did not change in composition between the young cones isolated from both the urbanised and natural sites. The lack of change in SFE communities in both habitats provides a positive overview of the impact of urbanisation on these microbial species within cities despite a small seasonal difference during collection. While seasonal effects may have confounded the comparison between urban and native sites, they did not appear to influence the outcome. What remains unclear is how quickly SFEs may vary seasonally, although it might be expected, given the woody infructescence acting as a barrier, to only occur slowly. However, the collection of samples from urban sites in the seasonally warm, wet, and humid weather of the summer might have led to greater isolation frequency and diversity of SFEs in comparison to collection carried out in the drier weather experienced during the spring. Precipitation during the collection period may increase fungal diversity and abundance [106]. Interestingly, we would expect that fungal frequency is also influenced by habitat size, density, and fragmentation [106,107]. Both RNP and PTG were large in area and consisted of relatively dense vegetation in comparison to the fragmented islands of LNP and BNP. In this case, we would expect to observe more airborne fungal spores, although the success of infection requires the correct conditions regarding rainfall, humidity, temperature, and windiness [107]. Future research into the rates of change of seed endophytes will provide important insights into the process of environmental transmission to woody infructescences.

Our results suggested some evidence of that competition within a seed over time results in a change in species composition, although further work is needed in this regard. Early-arriving SFEs may lose their place over time and become less abundant. Our culturing method tended to only identify one fungal endophyte per seed (occasionally two) and did not provide evidence of the presence of multiple endophytes in a seed, as slow-growing, unculturable, or rarer species may be quickly outcompeted on plates by competitive endophytes. Our isolates, therefore, represent the most competitive species within each seed. If competition between multiple endophyte species within a seed occurred over time, then we should have seen a similar assemblage amongst older seeds from both sites, as uncompetitive species would have disappeared, and assemblages should have grown more similar. There was some evidence of this in our study, suggesting some competition between endophytes might have occurred. There is the possibility that with age, the establishment of arriving endophytes may be influenced by those already present (a priority effect) [108]. Such patterns are currently poorly understood, and it is not known whether the host plants can provide a filter to influence which species are present.

This is the first study to document changes in seed endophyte assemblages with time providing evidence of changes that environmental transmission may cause and show a suite of patterns in transmission that need further investigation. This study identified that the ecology of endophyte communities is poorly understood, and with increasing urbanisation, there is a need to comprehensively understand how these endophytic species may influence plant communities.

## Figures and Tables

**Table 1:** Identity of OTUs representing cultured *Banksia ericifolia* seed fungal endophytes. Shaded squares show presence of different OTUs in seeds from trees in four different sites: two natural (Royal National Park and Patonga) and two urban sites (Lane Cove National Park and Kamay Botany Bay National Park). In native areas, old cones (greater than eight years) and young cones (less than four years) were sampled, while in urban areas, only young cones were sampled.

Species	OTU	PotentialFunctions	Royal NP		Patonga		Lane Cove NP	Botany NP
Cones			Old	Young	Old	Young	Urban	Urban
*Penicillium citreonigrum*	0	Saprotrophic, Pathogenic						
*Neofusicoccum hellenicum*	1	Saprotrophic, Pathogenic						
*Penicillium glabrum*	2	Saprotrophic, Pathogenic						
*Banksiamyces* sp.	3	Saprotrophic						
*Neocucurbitaria* sp.	4	Saprotrophic						
*Penicillium catalonicum*	5	Saprotrophic, Pathogenic						
*Cladosporium perangustum*	6	Saprotrophic						
*Exophiala bergeri*	7	Saprotrophic, Pathogenic						
*Cladophialophora mycetomatis*	8	Pathogenic						
*Talaromyces chlorolomus*	9	Saprotrophic, Pathogenic						
*Penicillium dierckxii*	10	Saprotrophic, Pathogenic						
*Penicillium* sp.	11	Saprotrophic, Pathogenic						
*Penicillium* sp.	12	Saprotrophic, Pathogenic						
*Sordariomycetes* sp.	13	Saprotrophic, Pathogenic						
*Neopestalotiopsis clavispora*	14	Pathogenic						
*Pestalotiopsis* sp.	15	Pathogenic						
*Anthostomelloides* sp.	16	Saprotrophic, Pathogenic						
*Paecilomyces maximus*	17	Saprotrophic, Pathogenic						
*Fusarium* sp.	18	Saprotrophic, Pathogenic						
*Penicillium olsonii or* sp.	19	Saprotrophic, Pathogenic						
*Cytospora eucalypticola*	20	Pathogenic						
*Colletotrichum endophyticum*	21	Saprotrophic, Pathogenic						
*Penicillium Sumatraense*	22	Saprotrophic, Pathogenic						
*Anteaglonium* sp.	23	Saprotrophic						
*Purpureocillium lilacinum*	24	Saprotrophic						
*Heleiosa barbatula*	25	Saprotrophic						
*Fimetariella rabenhorstii*	26	Pathogenic						
*Neofusicoccum parvum*	27	Pathogenic						
*Phaeoacremonium scolyti*	28	Pathogenic						
*Penicillium citrinum*	29	Saprotrophic, Pathogenic						
*Camaropella pugillus*	30	Saprotrophic						
*Xylomelasma* sp.	31	Saprotrophic						
*Anthostomelloides brabeji*	32	Saprotrophic, Pathogenic						
*Exophiala oligosperma*	33	Saprotrophic						
*Rasamsonia columbiensis*	34	Saprotrophic						
*Phialemonium* sp.	35	Saprotrophic, Pathogenic						
*Botryosphaeria stevensii*	36	Pathogenic						
*Phaeoacremonium argentinense*	37	Pathogenic						

**Table 2:** Two-factor permutational analysis of variance of the community composition of seed fungal endophytes amongst ^a^ young (less than four years old) and old (greater than 8 years old) cones across two sites (Patonga and Royal National Park); ^b^ urban sites (Lane Cove National Park and Kamay Botany Bay National Park) versus young cones at native sites (Patonga and Royal National Park). Statistically significant results at *p* < 0.05 are denoted by asterisks.

Source	df	MS	Pseudo-F	*p* (perm)
Old Cones versus Young Cones ^a^				
Age	1	30,438	18.598	0.001 *
Site	1	6965.4	4.2559	0.001 *
Age × Site	1	6432.5	3.9303	0.001 *
Error	34	1636.6		
Total	37			
Urban versus Native ^b^				
Habitat	1	16,148	4.3171	0.333 n.s
Site (Habitat)	2	3740.5	1.4886	0.121 n.s
Error	32	2512.7		
Total	35			

## Data Availability

The data that support this study are available in GenBank with accession numbers listed in Table S1.

## Supplementary Materials

The following supporting information can be downloaded at: https://www.mdpi.com/article/10.3390/jof9070706/s1, Table S1: Data availability and accession numbers for GenBank.
