# Supplementary material for: The Influence of Cone Age and Urbanisation on the Diversity and Community Composition of Culturable Seed Fungal Endophytes within Native Australian Banksia ericifolia L.f. subsp. ericifolia
Source: J Fungi (Basel). 2023 Jun 27;9(7):706. doi: 10.3390/jof9070706 (PMC10381327; doi:10.3390/jof9070706)
Supplement: Supplementary file 1 [file jof-09-00706-s001.zip › jof-2464684-supplementary.pdf]

Table S1. Data availability and accession numbers for GenBank.

Voucher specimen	GenBank accession	Isolates	Phylum	Class	Order	Family	Genus	Species	Collection
RBG 7419	OQ913762	316	Ascomycota	Eurotiomycetes	Eurotiales	Aspergillaceae	*Penicillium*	*citreonigrum*	Australia: Lane Cove, NSW
RBG 7420	OQ913763								Australia: Kamay Botany Bay, NSW
RBG 7421	OQ913764								Australia: Lane Cove, NSW
RBG 7422	OQ913765								Australia: Patonga, NSW
RBG 7423	OQ913766								Australia: Patonga, NSW
RBG 7424	OQ913767								Australia: Patonga, NSW
RBG 7425	OQ913768								Australia: Patonga, NSW
RBG 7426	OQ913769								Australia: Patonga, NSW
RBG 7427	OQ913770								Australia: Patonga, NSW
RBG 7428	OQ913771								Australia: Patonga, NSW
RBG 7429	OQ913772								Australia: Wattamolla, NSW
RBG 7430	OQ913773								Australia: Wattamolla, NSW
RBG 7431	OQ913774								Australia: Wattamolla, NSW
RBG 7432	OQ913775								Australia: Wattamolla, NSW
RBG 7433	OQ913776								Australia: Wattamolla, NSW
RBG 7434	OQ913777								Australia: Wattamolla, NSW
RBG 7435	OQ913778								Australia: Wattamolla, NSW
RBG 7436	OQ913779								Australia: Wattamolla, NSW
RBG 7437	OQ913780								Australia: Wattamolla, NSW
RBG 7438	OQ913781								Australia: Wattamolla, NSW
RBG 7439	OQ913782								Australia: Wattamolla, NSW

RBG 7440	OQ913783								Australia: Patonga, NSW
RBG 7450	OQ913793	100	Ascomycota	Dothideomycetes	Botryosphaeriales	Botryosphaeriaceae	*Neofusicoccum*	*hellenicum*	Australia: Lane Cove, NSW
RBG 7451	OQ913794								Australia: Kamay Botany Bay, NSW
RBG 7452	OQ913795								Australia: Wattamolla, NSW
RBG 7453	OQ913796								Australia: Kamay Botany Bay, NSW
RBG 7454	OQ913797								Australia: Kamay Botany Bay, NSW
RBG 7455	OQ913798								Australia: Patonga, NSW
RBG 7456	OQ913799								Australia: Patonga, NSW
RBG 7457	OQ913800								Australia: Patonga, NSW
RBG 7458	OQ913801								Australia: Patonga, NSW
RBG 7459	OQ913802								Australia: Patonga, NSW
RBG 7460	OQ913803								Australia: Patonga, NSW
RBG 7461	OQ913804	75	Ascomycota	Eurotiomycetes	Eurotiales	Aspergillaceae	*Penicillium*	*glabrum*	Australia: Kamay Botany Bay, NSW
RBG 7462	OQ913805								Australia: Kamay Botany Bay, NSW
RBG 7463	OQ913806								Australia: Lane Cove, NSW
RBG 7464	OQ913807								Australia: Kamay Botany Bay, NSW
RBG 7465	OQ913808								Australia: Lane Cove, NSW
RBG 7466	OQ913809								Australia: Kamay Botany Bay, NSW
RBG 7467	OQ913810								Australia: Wattamolla, NSW
RBG 7468	OQ913811								Australia: Wattamolla, NSW
RBG 7469	OQ913812								Australia: Patonga, NSW
RBG 7470	OQ913813	240	Ascomycota	Leotiomycetes	Helotiales	Helotiales fam incertae sedis	*Banksiamyces*	sp.	Australia: Wattamolla, NSW
RBG 7471	OQ913814								Australia: Patonga, NSW

RBG 7472	OQ913815								Australia: Patonga, NSW
RBG 7473	OQ913816								Australia: Patonga, NSW
RBG 7474	OQ913817								Australia: Patonga, NSW
RBG 7475	OQ913818								Australia: Patonga, NSW
RBG 7476	OQ913819								Australia: Patonga, NSW
RBG 7477	OQ913820								Australia: Patonga, NSW
RBG 7478	OQ913821								Australia: Wattamolla, NSW
RBG 7479	OQ913822								Australia: Wattamolla, NSW
RBG 7480	OQ913823								Australia: Wattamolla, NSW
RBG 7481	OQ913824								Australia: Wattamolla, NSW
RBG 7482	OQ913825								Australia: Kamay Botany Bay, NSW
RBG 7483	OQ913826								Australia: Kamay Botany Bay, NSW
RBG 7484	OQ913827								Australia: Lane Cove, NSW
RBG 7485	OQ913828								Australia: Lane Cove, NSW
RBG 7486	OQ913829								Australia: Lane Cove, NSW
RBG 7487	OQ913830								Australia: Lane Cove, NSW
RBG 7510	OQ913853	6	Ascomycota	Dothideomycetes	Pleosporales	Cucurbitariaceae	*Neocucurbitaria*	sp.	Australia: Wattamolla, NSW
RBG 7511	OQ913854	3	Ascomycota	Eurotiomycetes	Eurotiales	Aspergillaceae	*Penicillium*	*catalonicum*	Australia: Wattamolla, NSW
RBG 7448	OQ913791	1	Ascomycota	Dothideomycetes	Cladosporiales	Cladosporiaceae	*Cladosporium*	*perangustum*	Australia: Wattamolla, NSW
RBG 7449	OQ913792	1	Ascomycota	Eurotiomycetes	Chaetothyriales	Herpotrichiellaceae	*Exophiala*	*bergeri*	Australia: Wattamolla, NSW
RBG 7512	OQ913855	2	Ascomycota	Eurotiomycetes	Chaetothyriales	Herpotrichiellaceae	*Cladophialophora*	*mycetomatis*	Australia: Wattamolla, NSW
RBG7350	OP437839	1	Ascomycota	Eurotiomycetes	Eurotiales	Trichocomaceae	*Talaromyces*	*chlorolomus*	Australia: Wattamolla, NSW
RBG 7488	OQ913831	7	Ascomycota	Eurotiomycetes	Eurotiales	Aspergillaceae	*Penicillium*	*dierckxii*	Australia: Patonga, NSW

RBG 7489	OQ913832								Australia: Patonga, NSW
RBG 7490	OQ913833								Australia: Patonga, NSW
RBG 7491	OQ913834	4	Ascomycota	Eurotiomycetes	Eurotiales	Aspergillaceae	*Penicillium*	sp.	Australia: Patonga, NSW
RBG 7492	OQ913835								Australia: Patonga, NSW
RBG 7493	OQ913836	36	Ascomycota	Eurotiomycetes	Eurotiales	Aspergillaceae	*Penicillium*	sp.	Australia: Patonga, NSW
RBG 7494	OQ913837								Australia: Patonga, NSW
RBG 7495	OQ913838								Australia: Patonga, NSW
RBG 7496	OQ913839								Australia: Patonga, NSW
RBG 7497	OQ913840								Australia: Patonga, NSW
RBG 7441	OQ913784	13	Ascomycota	Sordariomycetes	N/A	N/A	N/A	N/A	Australia: Patonga, NSW
RBG7405	OP437894	3	Ascomycota	Sordariomycetes	Xylariales	Sporocadaceae	*Neopestalotiopsis*	*clavispora*	Australia: Patonga, NSW
RBG 7498	OQ913841	10	Ascomycota	Sordariomycetes	Xylariales	Sporocadaceae	*Pestalotiopsis*	sp.	Australia: Kamay Botany Bay, NSW
RBG 7499	OQ913842								Australia: Patonga, NSW
RBG 7500	OQ913843								Australia: Patonga, NSW
RBG 7442	OQ913785	1	Ascomycota	Sordariomycetes	Xylariales	Xylariales incertae sedis	*Anthostomelloides*	sp.	Australia: Patonga, NSW
RBG7347	OP437836	3	Ascomycota	Eurotiomycetes	Eurotiales	Thermoascaceae	*Paecilomyces*	*maximus*	Australia: Patonga, NSW
RBG 7513	OQ913856	2	Ascomycota	Sordariomycetes	Hypocreales	Nectriaceae	*Fusarium*	sp.	Australia: Patonga, NSW
RBG7346	OP437835	4	Ascomycota	Eurotiomycetes	Eurotiales	Aspergillaceae	*Penicillium*	*Olsonii*	Australia: Patonga, NSW
RBG 7514	OQ913857	2	Ascomycota	Sordariomycetes	Diaporthales	Valsaceae	*Cytospora*	*eucalypticola*	Australia: Patonga, NSW
RBG7364	OP437853	2	Ascomycota	Sordariomycetes	Glomerellales	Glomerellaceae	*Colletotrichum*	*endophyticum*	Australia: Patonga, NSW
RBG 7443	OQ913786	1	Ascomycota	Eurotiomycetes	Eurotiales	Aspergillaceae	*Penicillium*	*sumatraense*	Australia: Patonga, NSW
RBG 7501	OQ913844	3	Ascomycota	Dothideomycetes	Pleosporales	Anteagloniaceae	*Anteaglonium*	sp.	Australia: Kamay Botany Bay, NSW
RBG 7502	OQ913845								Australia: Wattamolla, NSW

RBG 7503	OQ913846								Australia: Wattamolla, NSW
RBG 7444	OQ913787	1	Ascomycota	Sordariomycetes	Hypocreales	Ophiocordycipitaceae	*Purpureocillium*	*lilacinum*	Australia: Wattamolla, NSW
RBG 7445	OQ913788	1	Ascomycota	Dothideomycetes	Pleosporales	Pleosporales incertae sedis	*Heleiosa*	*barbatula*	Australia: Wattamolla, NSW
RBG 7446	OQ913789	2	Ascomycota	Sordariomycetes	Sordariales	Lasiosphaeriaceae	*Fimetariella*	*rabenhorstii*	Australia: Wattamolla, NSW
RBG 7504	OQ913847	5	Ascomycota	Dothideomycetes	Botryosphaeriales	Botryosphaeriaceae	*Neofusicoccum*	*parvum*	Australia: Lane Cove, NSW
RBG 7505	OQ913848								Australia: Kamay Botany Bay, NSW
RBG 7506	OQ913849								Australia: Lane Cove, NSW
RBG 7314	OP437803								Australia: Lane Cove, NSW
RBG 7507	OQ913850	16	Ascomycota	Sordariomycetes	Togniniales	Togniniaceae	*Phaeoacremonium*	*scolyti*	Australia: Kamay Botany Bay, NSW
RBG 7508	OQ913851	11	Ascomycota	Eurotiomycetes	Eurotiales	Aspergillaceae	*Penicillium*	*citrinum*	Australia: Kamay Botany Bay, NSW
RBG 7509	OQ913852								Australia: Kamay Botany Bay, NSW
RBG 7515	OQ913858	1	Ascomycota	Sordariomycetes	Boliniales	Boliniaceae	*Camaropella*	*pugillus*	Australia: Lane Cove, NSW
RBG7393	OP437882	1	Ascomycota	Sordariomycetes	Sordariomycetidae	Sordariomycetidae incertae sedis	*Xylomelasma*	sp.	Australia: Kamay Botany Bay, NSW
RBG7406	OP437895	1	Ascomycota	Sordariomycetes	Xylariales	Xylariales incertae sedis	*Anthostomelloides*	*brabeji*	Australia: Lane Cove, NSW
RBG7334	OP437823	4	Ascomycota	Eurotiomycetes	Chaetothyriales	Herpotrichiellaceae	*Exophiala*	*oligosperma*	Australia: Kamay Botany Bay, NSW
RBG7348	OP437837	1	Ascomycota	Eurotiomycetes	Eurotiales	Trichocomaceae	*Rasamsonia*	*columbiensis*	Australia: Lane Cove, NSW
RBG 7447	OQ913790	1	Ascomycota	Sordariomycetes	Cephalothecales	Cephalothecaceae	*Phialemonium*	sp.	Australia: Kamay Botany Bay, NSW
RBG7313	OP437802	1	Ascomycota	Dothideomycetes	Botryosphaeriales	Botryosphaeriaceae	*Botryosphaeria*	*stevensii*	Australia: Lane Cove, NSW
RBG7394	OP437883	1	Ascomycota	Sordariomycetes	Togniniales	Togniniaceae	*Phaeoacremonium*	*argentinense*	Australia: Lane Cove, NSW
